# Skeletal interoception regulates joint homeostasis and PGE2-induced pain: implication of disease-modifying treatment

**DOI:** 10.1038/s41413-026-00561-1

**Published:** 2026-08-13

**Authors:** Qimiao Hu, Bonuo Qi, Yue Dong, Yushuang Pan, Yingjun Liu, Zhineng Chen, Jianqiao Fang, Yi Liang, Peng Zhang

**Affiliations:** 1 Department of Neurobiology and Acupuncture Research, The Third Clinical Medical College, Zhejiang Chinese Medical University, Hangzhou, China; 2 Zhejiang Chinese Medical University, Hangzhou, China; 3 The Third Clinical Medical College, Zhejiang Chinese Medical University, Hangzhou, China

**Keywords:** Neurophysiology, Bone

## Abstract

Interoception is a core process through which the body perceives its internal state and regulates physiological homeostasis via bidirectional communication between the central and peripheral nervous system. Skeletal interoception is a specific circuitry for the brain control of the weight-bearing system, particularly responsible for sensing bone-derived internal signals to maintain skeletal homeostasis in response to mechanical loading. Recent studies uncovered that prostaglandin E2 (PGE2) plays a crucial role in skeletal interoception, and is therefore involved in major skeletal disorders and pain conditions such as low back pain, osteoarthritis and particularly ankle osteoarthritis (AOA). Ankle pain is clinically common, with a prevalence of 9%-15% among adults, severely impairing work productivity and quality of life. This article reviews the progress of skeletal interoception in skeletal pathogenesis and pain, with AOA as an example. Specifically, it discusses PGE2 and skeletal interoception in relation to pain and inflammation. We also attempted to interpret non-steroidal anti-inflammatory drugs (NSAIDs), surgical interventions and Traditional Chinese Medicine (TCM) therapies, especially acupuncture and electroacupuncture, in the therapy of pain and osteoarthritis from the viewpoint of skeletal interoception. Interoception is an emerging science in understanding how the brain regulates peripheral organs. Skeletal interoception mediated by PGE2 provides an opportunity to understand the potential of NSAIDs and acupuncture in regulating interoception for the treatment of skeletal disorders including ankle pain.

## Introduction

The traditional view classifies the body’s perception of various stimuli into three categories: exteroception, which originates from external bodily signals; proprioception, which reflects the position and movement status of limbs and body parts; and interoception, which transmits information about the body’s internal states.^1,2^ According to some theories, proprioception and exteroception are combined^3^ and collectively referred to as somatosensation.^3^ Somatosensation is the body’s comprehensive ability to receive and transmit external physical stimuli or somatic mechanical state signals through sensory receptors located in the skin, muscles, joints, and other regions, encompassing tactile sensation, thermal sensation, nociception, as well as proprioceptive sensations such as position sense and kinesthesia.^4–6^

Interoception is defined as the perception of internal bodily states and plays a vital role in physical, cognitive, emotional, and social health.^7^ From a physiological perspective, interoception is realized through integrated bidirectional communication between the central nervous system and peripheral organs. It enables accurate perception and dynamic regulation of the body’s internal physiological and metabolic states, such as energy homeostasis, blood pressure regulation, intestinal peristalsis, and bone mineral density maintenance.^8^ The conduction of interoceptive signals follows a closed-loop process consisting of perception, transmission, integration and regulation. Firstly, interoceptors at the peripheral sensory nerve endings, which cover multiple neuron types such as humoral receptors, chemoreceptors, free nerve endings, specialized mechanoreceptors and nociceptors, receive four core types of signals: biochemical, mechanical, thermal and electromagnetic.^9,10^ Subsequently, these signals are converted into hormonal signals, electrical signals, or other non-neuronal signals, and relayed to the brain via two main ascending afferent pathways, the spinal nerves and the vagus nerves. Next, the signals are coordinately integrated and interpreted by multiple brain regions, including the nucleus tractus solitarius, thalamus, hypothalamus, parabrachial nucleus, hippocampus, anterior cingulate cortex, amygdala, superior colliculus, caudal periaqueductal gray, anterior insular cortex, prefrontal cortex, and orbitofrontal cortex. Ultimately, the brain dispatches regulatory signals back to the original signal-emitting organs via descending efferent pathways, completing homeostatic regulation.^11^

The skeletal system, as a vital organ of the human body, not only provides mechanical supportive function for the body to adapt to ground movement but also plays an important role in regulating systemic metabolism.^12^ Mechanical stimulation is the most common extrinsic stimulus to the skeleton, which adapts to mechanical loading via the dynamic balance between bone resorption and bone formation. And studies have found that mechanical signals participate in the brain’s regulation of the skeleton through skeletal interoception.^13^ Skeletal interoception is specifically characterized as the perception and processing of skeletal signals, an independent branch of interoception, which plays a vital role in sustaining skeletal homeostasis and mediating responses to mechanical loading.^12^ The interoceptive capacity is modulated by specialized bone tissue cells such as osteoblasts, osteoclasts, and osteocytes. These cells can perceive physical pressure and mechanical stress, and then regulate bone metabolism through a series of physiological mechanisms.^10^ The skeleton is innervated by an abundance of sensory and autonomic nerves, which are widely distributed in bone tissue and on joint surfaces and can relay interoceptive signals to the central nervous system. As an important part of the central nervous system, the hypothalamus can process these interoceptive signals and regulate skeletal homeostasis via the autonomic nervous system, neuroendocrine mechanisms, and neuropeptide release.^14^

Skeletal interoception relies on two types of neural pathways, ascending and descending, that combine to constitute a complete feedback loop. The ascending pathways are responsible for transmitting interoceptive signals from the skeleton to the central nervous system via two main routes. The first route involves the central processes of dorsal root ganglion (DRG) neurons projecting to the spinal dorsal horn, where signals are integrated and transmitted to the central nervous system via the spinal dorsal columns. The second route relies on the jugular and nodose ganglia of the vagus nerve, which transduce related interoceptive signals to the brainstem. However, the role of these ganglia in skeletal signal transmission remains unconfirmed; only the synapse formation of vagal afferent nerves within the NTS has been established. The descending pathways, on the other hand, are a crucial component of the functional interoceptive loop. After the central nervous system integrates the incoming interoceptive inputs, it orchestrates involuntary efferent responses primarily via the autonomic nervous system, transmitting centrally generated signals back to peripheral target organs to regulate organ function and sustain homeostasis.^14^

In the signal transmission of skeletal interoception, prostaglandin E2 (PGE2) serves the role of a core mediator and also a biochemical indicator of skeletal mechanical loading.^13,15^ It not only mediates the detection of variations in bone mineral density but also serves as a key trigger for the regulation of skeletal interoception’s sensitivity to mechanical stimuli and energy balance, among which the PGE2/EP4 signaling pathway plays a central role.^13^ Specifically, when bones are stimulated by mechanical loading, osteoblasts secrete PGE2. PGE2 can activate the EP4 receptor in ascending sensory nerves, which in turn triggers signal processing in the hypothalamus and stimulating CREB signaling phosphorylation in the ventromedial hypothalamic nucleus (VMH). Phosphorylated CREB downregulates the tone of descending sympathetic nerves, ultimately enhancing the differentiation of bone marrow mesenchymal stem cells (BMSCs) toward osteoblasts and augmenting osteogenesis. This regulatory cascade not only maintains bone homeostasis but also accelerates bone regeneration. Moreover, this activated PGE2/EP4 skeletal interoceptive ascending pathway can modulate the neuroendocrine system to suppress neuropeptide Y (NPY) expression in the arcuate nucleus (ARC) of the hypothalamus. This further facilitates lipolysis in white adipose tissue and bone marrow adipose tissue, leading to the release of free fatty acids that offer support for osteogenesis and further synergistically sustains the homeostasis of bone remodeling and BMSCs differentiation.^9^ Thus, skeletal interoception relies on the bone central neural feedback loop and uses the PGE2/EP4 signaling pathway as the core mediating pathway to achieve coordinated regulation of bone homeostasis. Table 1 below summarizes the comparisons of key steps in skeletal interoceptive signal transduction.

**Table 1:** Comparison of key links in skeletal interoceptive signal conduction

Signal transduction step	Core participating structures/molecules	Normal physiological process	References
Peripheral signal perception	Osteoblasts, EP4 receptors (sensory nerve endings)	Mechanical loading → PGE2 secretion by osteoblasts → EP4 receptor activation → Sensory signaling initiation	^9^
Signal conversion (peripheral → central)	DRG neurons	Receptor activation → DRG electrical signal → spinal dorsal horn input	^9^
Central signal integration	VMH, CREB Protein	Spinal dorsal horn signal → VMH CREB phosphorylation → Sympathetic tone downregulation	^9,16^
Signal regulation (central → peripheral)	Sympathetic efferent fibers, ARC, NPY	1. Sympathetic downregulation → BMSCs osteogenic differentiation → Bone homeostasis maintenance 2. NPY → Adipose tissue lipolysis → Osteoblast-mediated bone formation	^9,16^

## Skeletal interoception regulation of weight bearing bone

**Fig. 1:**
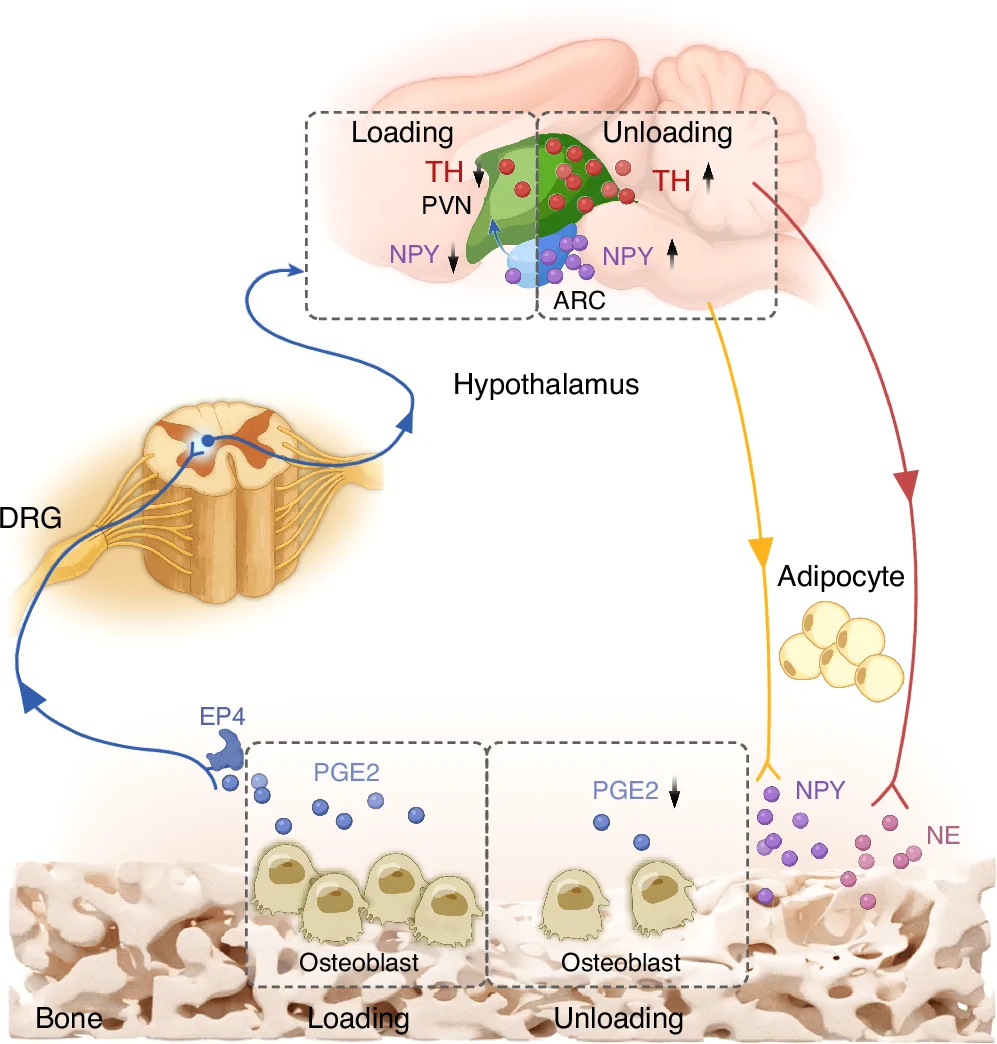
Skeletal interoception regulation of weight bearing bone. Mechanical loading can induce osteoblasts to secrete PGE2, which binds to and activates EP4 receptors on sensory nerves. Subsequently, signals are transmitted from the ARC to the PVN of the hypothalamus, thereby leading to the downregulation of TH transcriptional activity. In contrast, under unloading conditions, the PGE2-mediated ascending interoceptive signals within the hypothalamus are attenuated, resulting in a marked increase in the expression levels of NPY and TH in the hypothalamus. Elevated TH expression further enhances sympathetic tone. DRG Dorsal Root Ganglion, PVN Paraventricular Nucleus of the Hypothalamus, ARC Arcuate Nucleus of the Hypothalamus, TH Tyrosine Hydroxylase, NPY Neuropeptide Y, NE Norepinephrine

As a mechanosensitive tissue, bone relies on mechanical loading to preserve its homeostasis. Skeletal interoception can convert the mechanical signals of weight bearing bone into biochemical signals within osteocytes. Among them, PGE2 acts as a key mechanotransduction signal, mediating the conversion of external mechanical load into biochemical reactions. Mechanical loading increases bone PGE2 concentrations to regulate bone formation, while the hypothalamus senses this PGE2 elevation to modulate bone remodeling and skeletal structure. Specifically, mechanical loading can activate the skeletal interoceptive PGE2/EP4-ARC-PVN ascending pathway from bone to brain, and inhibit sympathetic tone by upregulating the phosphorylation level of CREB in the ARC of the hypothalamus, ultimately achieving precise regulation of bone remodeling.^16^ Under unloading or microgravity conditions, PGE2-mediated ascending interoception signals in the hypothalamus are weakened, leading to a significant increase in the expression of NPY and tyrosine hydroxylase (TH) in the hypothalamus. Among these, the increased TH expression enhances sympathetic tone, and the combined effect of high NPY expression and elevated sympathetic tone ultimately induces bone loss and adipose metabolic disorders.^17^ Thus, skeletal interoceptive can convert mechanical load signals of weight bearing bone into biochemical signals via the PGE2/EP4 signaling pathway and transmit them to the hypothalamus, thereby achieving precise regulation of bone remodeling; whereas the attenuation of this pathway under unloading or microgravity conditions can induce bone loss and adipose metabolism disorders (Fig. 1).

### Skeletal interoception regulation of subchondral bone

The subchondral bone is located beneath the articular cartilage and is responsible for absorbing, dispersing, and transmitting the mechanical loads borne by the joint. Within the subchondral bone tissue, the osteoblast lineage serves as the primary source of PGE2 secretion.^18^ Excessive PGE2 expression can induce pathological remodeling of the subchondral bone, thereby altering the mechanical stress distribution pattern of the overlying articular cartilage.^19^

Specifically, the PGE2/EP4 signaling pathway in osteoclasts mediates H-type angiogenesis and calcitonin gene-related peptide-positive (CGRP^+^) sensory neuron innervation in the subchondral bone.^18^ On the one hand, after PGE2 binds to EP4 receptors on sensory nerves, it regulates sympathetic nerve activity through the central nervous system, which in turn affects the bone-forming function of osteoblasts. On the other hand, H-type blood vessels themselves have the ability to regulate bone formation.^20^ Therefore, elevated PGE2 levels in the subchondral bone simultaneously promote enhanced local sensory innervation and increased H-type angiogenesis. These dual effects amplify the aforementioned pathological processes, ultimately leading to pathological changes in the subchondral bone structure. As the supportive substrate of articular cartilage, any structural abnormalities of the subchondral bone will directly disrupt the mechanical stress distribution on the surface of the overlying cartilage, resulting in localized stress concentration.^21^

### Skeletal interoception regulation of articular cartilage

**Fig. 2:**
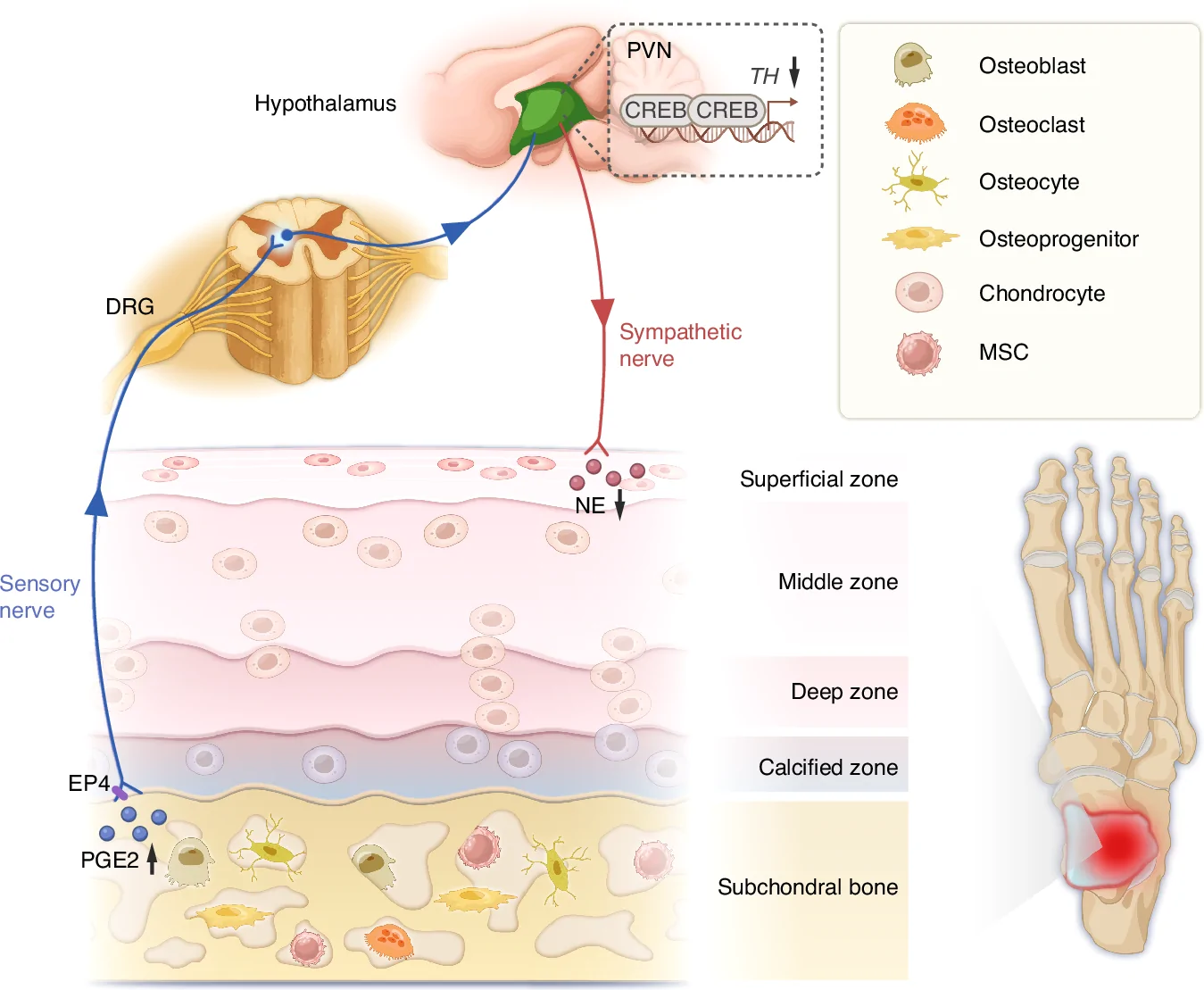
Skeletal interoception regulation of Articular cartilage. Mechanical loading potentiates PGE2-mediated skeletal interoception, which in turn downregulates NE levels to facilitate anabolic renewal of the superficial membrane and thereby preserve the appropriate thickness and structural integrity of articular cartilage. CREB cAMP Response Element-Binding Protein, TH Tyrosine Hydroxylase, NE Norepinephrine

Articular cartilage is a thin layer of connective tissue within diarthrodial joints, devoid of nerves and blood vessels. It provides a lubricated surface for low-friction movement and transmits mechanical load to the subchondral bone. Joint friction readily causes damage to the superficial layer of cartilage, and chondrocytes in the superficial zone (SFZ) enable dynamic regeneration and repair of the superficial layer via anabolic matrix synthesis. This process is regulated by skeletal interoception mediated by the hypothalamus through PGE2 (Fig. 2). Specifically, mechanical load stimulates osteoblasts to secrete PGE2, which binds to the EP4 receptor on sensory nerves, initiating skeletal interoceptive signals that project to the PVN of the hypothalamus and subsequently activate the central CREB signaling pathway. This activation reduces sympathetic tone, inhibits the expression of TH, a key enzyme for norepinephrine (NE) synthesis in the PVN, and decreases NE release from sympathetic nerve terminals. In turn, this regulates the anabolism of the superficial layer, modulates the expression of transforming growth factor beta (TGF-β) and lubricin in the superficial zone, and maintains the structural integrity of the deep and calcified zones of cartilage.^22^

Among these factors, Lubricin is secreted by chondrocytes in the superficial zone and is essential for the structure and regenerative activity of articular cartilage. Previous studies have confirmed that TGF-β upregulates lubricin expression in articular chondrocytes at the transcriptional level. Enhanced skeletal interoception reduces sympathetic tone and NE release, which directly promotes lubricin secretion by superficial zone chondrocytes and indirectly upregulates lubricin expression by activating the TGF-β signaling pathway. Diffused lubricin further stimulates matrix protein synthesis in the deep zone of cartilage.^22^

Overall, mechanical loading can promote the regeneration and repair of the superficial articular cartilage layer and maintain articular cartilage health by enhancing the PGE2/EP4 signaling pathway and downregulating the level of hypothalamus-derived NE.

### Aberrant mechanical loading affects skeletal interoception to cause joint disorders and pain

Aberrant mechanical loading can stimulate osteoclasts in the vertebral endplates, subchondral bone of articular cartilage, or bones in other regions to perform bone resorption, resulting in the formation of porous structures.^16^ In porous bone tissue, Netrin-1 released by the bone matrix and osteoclasts promotes the neural innervation mediated by CGRP-expressing (CGRP⁺) neurons, exacerbating OA and other related pains.^9^ Additionally, this process also leads to reduced bone mineral density and elevated PGE2 concentrations. High levels of PGE2 trigger inflammatory hyperalgesia and sensory neuronal sensitization via the EP4 receptor, and activate nociceptive ion channels including TRPV1 and Nav1.8, ultimately generating pain signals.^9^ To summarize, aberrant mechanical loading disrupts skeletal interoceptive by inducing porous bone remodeling, promoting nociceptive innervation, and elevating PGE2 levels, ultimately triggering joint disorders and pain.

As a therapeutic intervention, downregulating pathologically elevated PGE2 levels in skeletal tissues to their physiological range can activate skeletal interoception and induce osteoblastic bone formation. Specifically, this process reduces vertebral endplate porosity, which in turn alleviates low back pain and ultimately delays the progression of diseases such as OA.^23^ For example, recent studies have confirmed that low-dose celecoxib can enhance osteogenesis within porous vertebral endplates by sustaining PGE2 concentrations within the physiological range.^24^ Therefore, maintaining PGE2 at its physiological level is crucial.

## Correlations between PGE2 and Skeletal Diseases

We systematically summarize the research progress of related skeletal disorders from the perspective of PGE2 in Table 2. In recent studies, the PGE2-mediated skeletal interoception pathway has attracted extensive attention in diseases such as OA, Low Back Pain (LBP), Lumbar Spine Instability (LSI), Postmenopausal Osteoporosis (PMOP) and Rheumatoid Arthritis (RA). These studies have confirmed that above-mentioned diseases are closely associated with the regulation of PGE2 and its EP receptors. For RA, an autoimmune disease, recent research has focused more on the regulatory effect of PGE2 on immune cells. Overall, the correlation between PGE2 and the aforementioned diseases has been preliminarily verified.

**Table 2:** Correlations between PGE2 and skeletal disorders

Disorder	Disorder definition	Characteristics	PGE2-related mechanisms	References
Osteoarthritis (OA)	A joint degenerative disease	Cartilage degeneration and calcification, synovial inflammation, and subchondral bone remodeling	PGE2/EP4 signaling in osteoclasts mediates angiogenesis and sensory neuron innervation in subchondral bone, promoting OA progression and pain Excessive PGE2 in subchondral bone caused a pathological alteration to subchondral bone in OA	^18,19,111^
Low back pain (LBP)	A chronic pain syndrome	Nociceptive, neuropathic, and central sensitization	Abnormal PGE2 concentrations in endplate activate EP4 causing sensory hypersensitivity and LBP	^9,112,113^
Lumbar spine instability (LSI)	A significant spinal disorder	Abnormal strains or excessive motion occur in the functional spinal unit (including upper/lower vertebral bodies, IVD, and facet joints)	Elevated PGE2 in porous endplates induces sodium influx and sensory nerve activation leading to spinal pain in LSI PGE2/EP4 pathway in porous endplates activates DRG TRPV1 channels leading to spinal hypersensitivity in LSI	^30,114,115^
Postmenopausal osteoporosis (PMOP)	A systemic bone disease	Reduced bone mass and the deterioration of bone microarchitecture	Activation of PGE2 Receptor EP4 induces bone remodeling Loss of the PGE2 Receptor EP1 enhances bone acquisition	^116–118^
Rheumatoid arthritis (RA)	A chronic, systemic autoimmune disease	Synovial tissue proliferation, pannus formation, cartilage destruction, systemic complications	PGE2 promotes IL-17A production and CD80/CD86 expression in γδ T cells of RA patients PGE2/IL-23 link STAT3–RORγ to drive Th17 CD4⁺ T-cell development in RA	^119–121^

### PGE2 in skeletal pain

PGE2 is a core inflammatory mediator of skeletal pain.^25^ PGE2 at physiological concentrations plays a vital role in maintaining bone homeostasis, whereas under pathological conditions, local inflammation or injury caused by fractures, OA, bone tumors and other factors induces a surge in PGE2 synthesis, thereby triggering pain signals.^26,27^ Recent evidence suggests that PGE2 is also involved in neural mechanisms, particularly peripheral sensitization. Peripheral sensitization is described as hyperexcitability of peripheral nociceptors, which is primarily attributed to the roles of neurotrophic factors and pro-inflammatory cytokines in promoting nociceptor depolarization.^28^

**Fig. 3:**
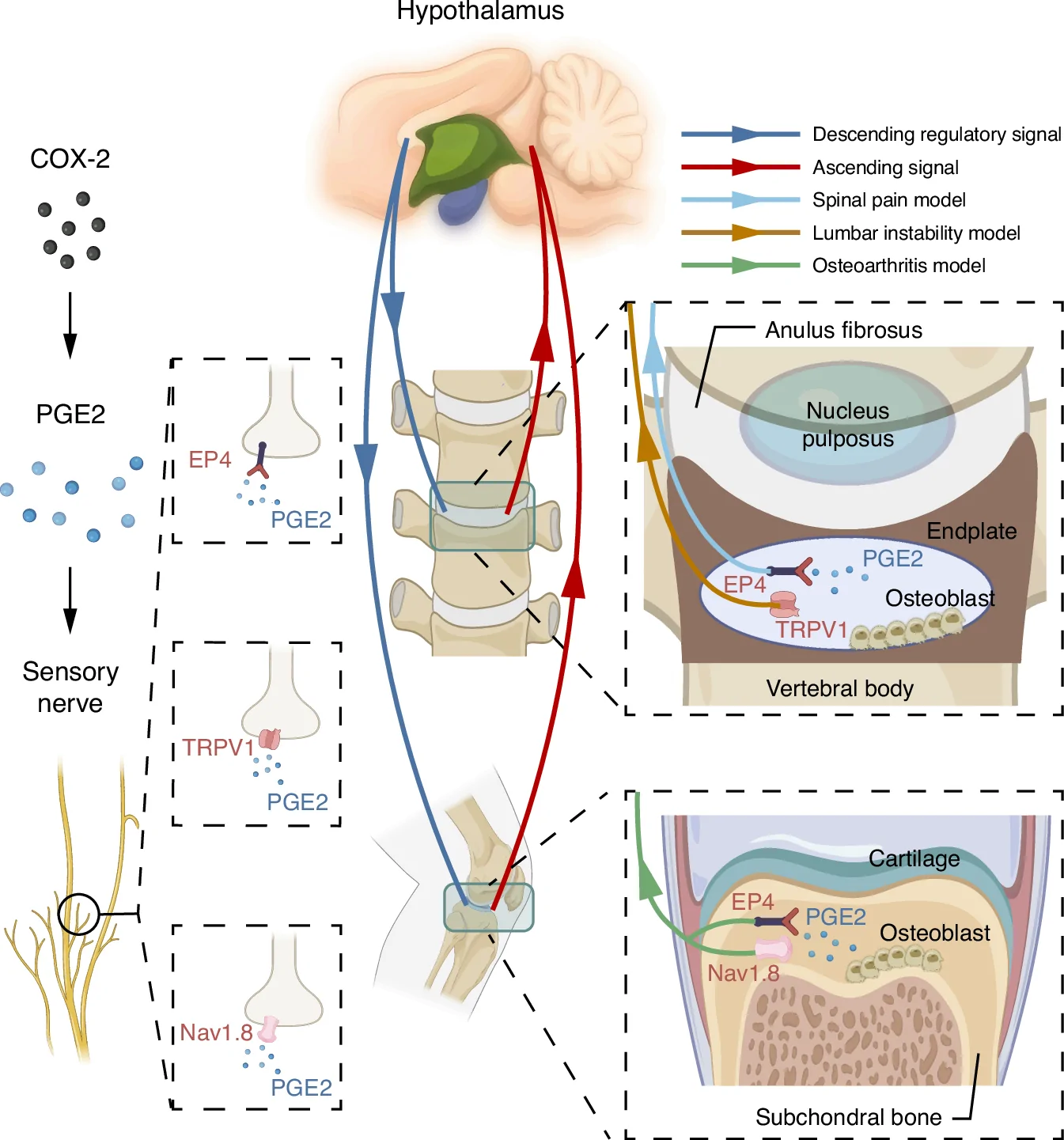
PGE2 in skeletal pain. PGE2 directly sensitizes peripheral nociceptors via EP4 receptors, TRPV1, and Nav1.8 to induce pain. In spinal pain, elevated PGE2 activates EP4 receptors on sprouting nerve fibers to trigger pain. For OA-associated pain, increased subchondral bone PGE2 stimulates EP4 receptors and Nav1.8 channels, causing sensory nerve hypersensitivity and exacerbating pain progression and maintenance. In a lumbar instability mouse model, elevated PGE2 activates TRPV1 channels in DRG neurons via EP4 receptors, enhancing spinal hyperalgesia

PGE2 can directly sensitize peripheral nociceptors by targeting EP4 receptors, TRPV1 and Nav1.8, thereby inducing pain (Fig. 3).^29^ On the one hand, PGE2 triggers inflammatory hyperalgesia and sensory neuronal sensitization through the EP4 receptor. For example, in the setting of spinal pain, elevated levels of PGE2 and increased density of sensory neuronal innervation are commonly observed in both porous and sclerotic vertebral endplates; moreover, PGE2 can induce spinal pain by specifically activating EP4 receptors on sprouting nerve fibers.^24^ Furthermore, during the onset of low back pain and OA-associated pain, the abnormal expression of PGE2 in the endplates and subchondral bone can elicit sensory nerve hypersensitivity by stimulating EP4 receptors, thereby exacerbating the progression and maintenance of such pain.^9,11^ On the other hand, the PGE2 signaling pathway can trigger the activation of various nociceptive ion channels, such as inducing calcium or sodium influx via TRPV1, or acting on the Nav1.8 channel.^24^ Specifically, in the lumbar instability mouse model, elevated PGE2 levels activated TRPV1 channels in DRG neurons via the EP4 receptor, leading to enhanced spinal hyperalgesia.^30^ Notably, as a member of the glial cell line-derived neurotrophic factor family ligands (GFLs), artemin (ARTN) can upregulate TRPV1 ion channels in DRG neurons via the p38 MAPK signaling pathway, and cooperate with the PGE2 pathway that directly activates TRPV1 to participate in the transmission and amplification of pain signals.^31,32^ Another study indicates that PGE2 sensitizes DRG neurons by modifying the Nav1.8, and genetic or pharmacological suppression of Nav1.8 channels in DRG neurons can considerably alleviate OA-associated pain.^25^ Collectively, these findings demonstrate that PGE2 participates in neural mechanisms of pain by mediating peripheral sensitization through binding to its receptor EP4 and activating nociceptive ion channels including TRPV1 and Nav1.8, thereby contributing to pain pathogenesis.

### Elevated PGE2 stimulates inflammation in arthritis

PGE2 can regulate the local joint microenvironment through multiple pathways to promote the occurrence and development of inflammatory responses.^33^ For instance, PGE2 can promote the release of pro-inflammatory cytokines. High concentrations of PGE2 can regulate the production of IL-6 in various cell types including macrophages and synovial fibroblasts and promote the secretion of IL-33 by macrophages.^34,35^ Furthermore, PGE2 induces local vasodilation in joints by binding to EP2 and EP4 receptors, increasing local blood flow and vascular permeability.^36^ Elevated vascular permeability leads to the extravasation of plasma proteins and inflammatory cells into the joint cavity, triggering synovial congestion and edema, and thus providing a material basis for the inflammatory response. Although EP3 receptor activation can antagonize this effect by enhancing endothelial barrier function, PGE2-mediated vascular hyperpermeability dominates in the pathological state of arthritis, which is a key characteristic of the early inflammatory response.^36^ In addition, PGE2 can directly inhibit proteoglycan synthesis in chondrocytes via the EP4 receptor, while enhancing the expression levels of matrix metalloproteinases such as MMP13 to promote cartilage matrix degradation, disrupting cartilage homeostasis, and thereby exacerbating joint inflammation and tissue damage.^37,38^ In summary, PGE2 jointly promotes the inflammatory progression and pathological damage of arthritis through diverse mechanisms, such as promoting the release of pro-inflammatory cytokines, regulating vascular function and disrupting cartilage homeostasis.

### Elevated PGE2 induces OA-associated pain

OA is characterized by persistent low-grade inflammation, with the innate immune system serving as the primary mediator, thereby triggering cartilage deterioration, bone remodeling, and synovial changes.^39^ In the pathological state of OA, the elevated PGE2 induces inflammatory pain through a multi-step cascade reaction. First, PGE2 has diverse cellular sources, primarily encompassing MSC-derived cells such as osteoblasts, osteocytes and chondrocytes, as well as myeloid cells including CD68⁺ macrophages, osteoclasts and other related subtypes.^28^ Among them, osteoblasts are the major releasers of PGE2 in the subchondral bone region and bone injury sites of OA.^19^ Additionally, pro-inflammatory cytokines including IL-1β and TNF-α can further induce an increase in PGE2 production.^40,41^ Meanwhile, PGE2 can upregulate COX-2 expression by activating the EP4 receptor on osteoblasts, thereby forming a COX-2/PGE2/EP4 positive feedback loop that drives further amplification of PGE2 synthesis.^19^ Subsequently, during the initiation of pain signals and peripheral sensitization, PGE2 can directly act on peripheral nociceptors in joint tissues, activating EP4 receptors and TRPV1. It can also promote the externalization of voltage-gated sodium channel Nav1.8, increase the surface density and activity of receptor cells, and lower the neural excitation threshold.^28^ Additionally, after binding to EP4 receptors on osteoclasts, PGE2 stimulates osteoclasts to release NGF and Netrin-1.^28^ This induces abnormal infiltration of sensory nerves into the subchondral bone and increases sensory nerve density. The pathway also promotes subchondral bone angiogenesis, amplifying the local inflammatory microenvironment.^18^ Finally, the sustained increase in nociceptive input from the periphery further leads to central sensitization in the dorsal horn of the spinal cord.^42^ In recent years, multiple studies have shown that inhibiting the COX-2/PGE2 pathway can alleviate the inflammatory response and associated pain in OA.^40,41,43–45^

### Acupuncture, electroacupuncture and skeletal interoception

**Fig. 4:**
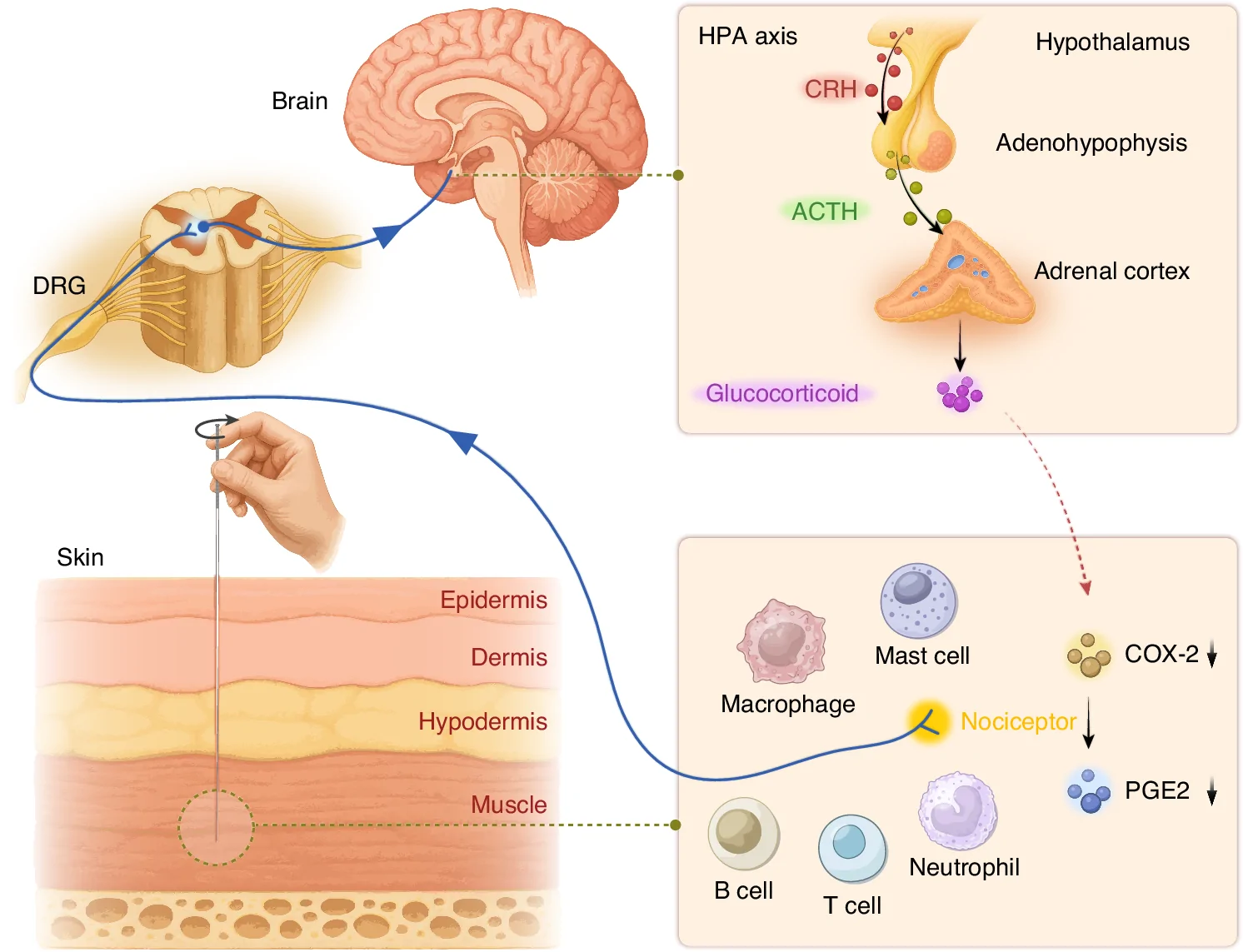
Acupuncture and the HPA axis. Acupuncture modulates the HPA axis and reduces peripheral COX-2/PGE2 levels to exert anti-inflammatory and analgesic effects. HPA hypothalamic-pituitary-adrenal, CRH Corticotropin-Releasing Hormone, ACTH Adrenocorticotropic Hormone

Acupuncture refers to a therapeutic method that stimulates specific acupoints on the human body by inserting thin metal needles and applying manipulations. It has been proven to be a viable therapeutic strategy for pain alleviation with extremely low risks. Recent studies have demonstrated that targeted regulation of hypothalamic nuclei is the core mechanism underlying acupuncture’s multiple physiological effects. Specifically, acupuncture can modulate the activity of the hypothalamic-pituitary-adrenal (HPA) axis by acting on the paraventricular nucleus (PVN), thereby downregulating the expression levels of COX-2 and PGE2 (Fig. 4); its therapeutic effect on osteoporosis in ovariectomized (OVX) rats is also closely associated with the regulation of HPA axis function.^46^ Notably, acupuncture significantly increased bone mass in rats with intact arcuate nucleus (ARC) but failed to improve that in lesioned ones, indicating ARC is an essential link for acupuncture to regulate bone mass.^47^ In addition, acupuncture-mediated regulation of the ventromedial hypothalamic nucleus (VMH) can suppress sympathetic nerve excitability, promote browning of white adipose tissue (WAT), and thus improve lipid metabolism.^48^ Meanwhile, the synergistic regulation of ARC and PVN by acupuncture can mediate central analgesic effects.^49^ Electroacupuncture is a form of acupuncture that achieves stimulation by applying a weak electric current between a pair of acupuncture needles. In recent years, studies have confirmed that electroacupuncture can exert analgesic effects through the DRG. Specifically, electroacupuncture exerts an inhibitory effect on the expression of PKCε and TRPV1 in the DRG.^50^ Additionally, when targeting the Yanglingquan (GB34) and Xiyan (EX-LE4) acupoints, electroacupuncture can significantly decrease the concentrations of pain-related factors such as PGE2, CGRP, and SP in the DRG and spinal dorsal horn, thereby alleviating spinal hyperalgesia in rats with knee osteoarthritis.^51^ These studies have revealed the close regulatory relationship between electroacupuncture and the DRG, and clarified that the downregulation of PGE2 levels is one of the important mechanisms mediating the analgesic effects of electroacupuncture. These findings also suggest that acupuncture and electroacupuncture may be associated with skeletal interoception, providing a crucial research direction for further exploring the molecular mechanisms through which acupuncture and electroacupuncture regulate skeletal interoception and expanding their application in bone-related pain disorders.

## Ankle pain

The ankle joint bears the highest body weight,^52^ and its associated pain syndromes significantly compromise locomotor function and quality of life.^53^ Ankle pain is a major feature of many diseases. Ankle pain may arise from ankle osteoarthritis (AOA), lateral ankle sprain (LAS), chronic ankle instability (CAI), etc. Notably, poor recovery from a LAS is likely to progress to CAI, and long-standing CAI can induce AOA.^54,55^ Ankle pain poses heavy burdens to both of the health-care services and suffering patients. Epidemiological investigations demonstrate that the prevalence of ankle pain among adults ranges from 9% to 15%, and ankle pain or injury accounts for as much as 45% of sports-associated injuries.^56^ And a cross-sectional survey based on community-dwelling elderly populations showed that the prevalence of ankle pain was relatively higher among female groups and individuals engaged in daily physical labor.^57^ Furthermore, chronic foot and ankle pain also imposes a substantial economic burden. For ankle sprains alone, the treatment cost per episode ranges from $292 to $2 268, while the annual medical expenditure associated with foot and ankle surgeries amounts to as much as 11 billion US dollars.^58^ Those with chronic pain also have a higher likelihood of experiencing emotional dysregulation, resulting in anxiety, fear, depressive symptoms, or even suicidal thoughts.^59^ Therefore, ankle pain exerts profound adverse effects on both the physical and mental aspects of patients’ lives.

At present, the treatment of ankle pain, is centered on relieving symptoms, mainly including pharmacological treatments such as nonsteroidal anti-inflammatory drugs (NSAIDs), physical therapy, and traditional Chinese medicine (TCM) therapies like acupuncture.^58,60–64^ However, NSAIDs exhibit suboptimal efficacy in the management of neuropathic pain and are prone to inducing adverse reactions including allergies, as well as gastrointestinal, renal and cardiovascular system disorders. This limitation significantly restricts their long-term clinical application.^65–67^ Currently, there is a lack of disease-modifying therapies for joint pain disorders, such that surgical interventions become the sole treatment modality for the advanced stage of these conditions.^58,60,68^ Therefore, understanding the mechanisms underlying ankle pain is imperative for identifying novel therapeutic targets.

### Elevated PGE2 is correlated with AOA and pain

AOA is a common chronic degenerative disease clinically characterized by cartilage degeneration, subchondral bone sclerosis, marginal osteophyte formation, and joint deformity, with the main manifestations of chronic pain and joint deformity.^69^ Notably, recent studies have found that the abnormally elevated level of PGE2 in the talus subchondral bone is associated with ankle-specific pain, and relevant evidence has been obtained from both AOA mouse models and talar samples of patients with end-stage AOA.

As the bone bearing the greatest body weight in humans, the talus exhibits a higher basal PGE2 concentration than other bones under physiological conditions. Aberrant mechanical loading induced by AOA leads to a marked upregulation of COX2 in the subchondral bone of talus. As the key rate-limiting enzyme for PGE2 synthesis, elevated COX2 expression directly results in abnormal accumulation of PGE2. In the AOA state, the abnormal PGE2 accumulation is accompanied by a significant increase in the density of CGRP⁺ sensory nerve fibers, which mediate pain signaling. PGE2 transduces signals via EP4, its key receptor on sensory nerves, thereby activating these CGRP⁺ sensory nerve fibers to transmit pain signals to the central nervous system, ultimately leading to ankle-specific pain. In addition, the subchondral bone of talus is characterized by an almost complete absence of osteoclast-mediated bone resorption. In the AOA state, the expression of cathepsin K (CTSK) secreted by osteocytes in this region is significantly upregulated, and CTSK directly exacerbates talar bone tissue damage, further providing a pathological basis for pain signal transmission. Collectively, the abnormal elevation of PGE2 combined with CTSK-mediated bone tissue damage contributes to the ankle-specificity of the resulting pain.^16^

## Therapeutic strategies for ankle pain

**Fig. 5:**
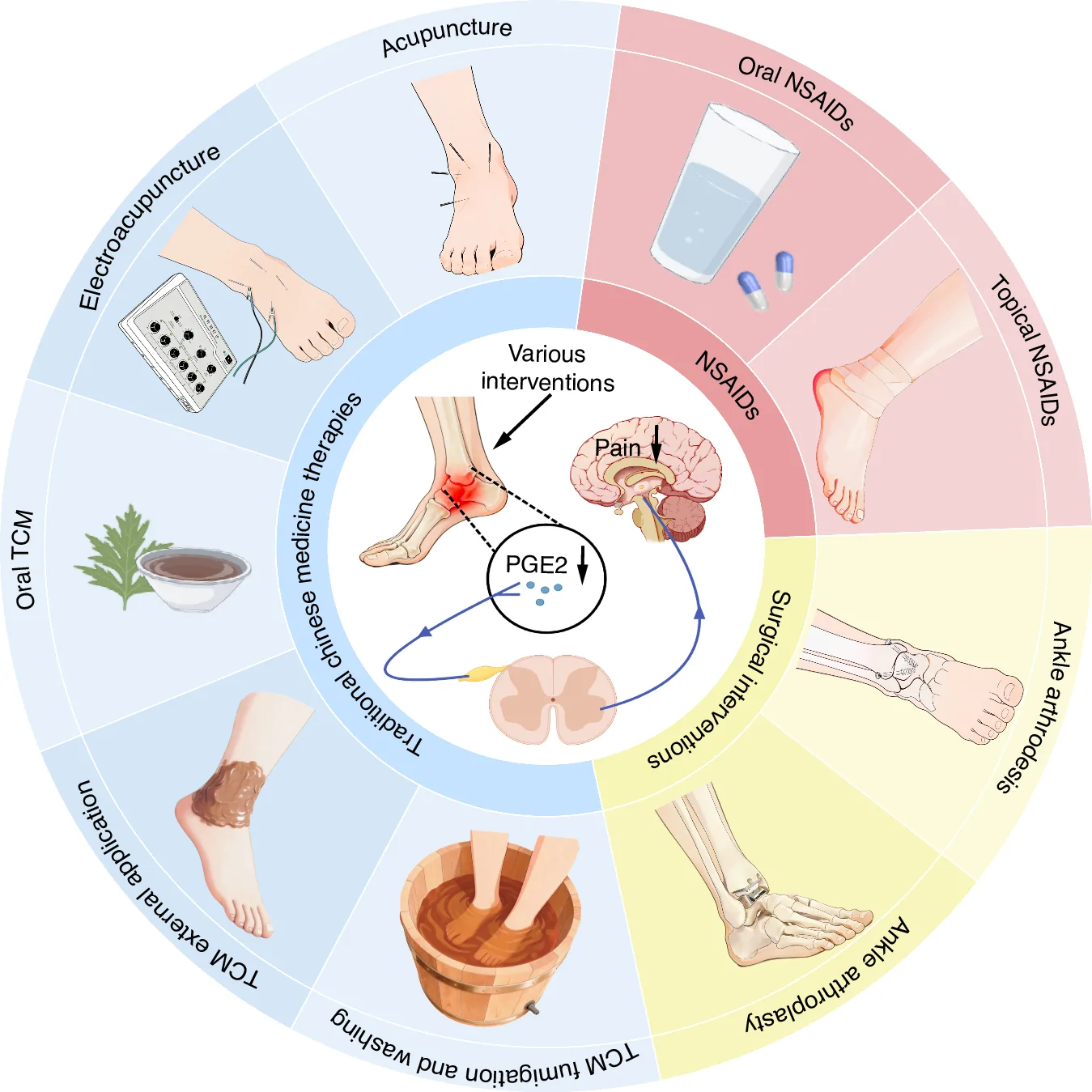
Therapeutic strategies for ankle pain. This figure illustrates multiple therapeutic strategies for ankle pain, including NSAIDs, TCM therapies, and surgical interventions, all of which aim to reduce PGE2 level to relieve pain

This article comprehensively reviews the current conventional therapeutic strategies for ankle pain in Western and TCM, which mainly consist of NSAIDs, acupuncture, electroacupuncture, oral TCM, TCM fumigation and washing, TCM external application, as well as surgical interventions (Fig. 5).

### Nonsteroidal anti-inflammatory drugs (NSAIDs) for ankle pain

NSAIDs exert excellent anti-inflammatory and analgesic effects and are widely used for musculoskeletal pain following trauma or surgery.^70^ The mechanistic basis of their effects involves inhibiting COX to block the production of prostaglandins.^71^ Notably, a recent study has confirmed an association between NSAIDs and skeletal interoception. In the mouse model of lumbar instability, low-dose celecoxib can inhibit COX-2 to regulate PGE2 to physiological concentrations; it may further maintain skeletal interoceptive homeostasis, reduce vertebral endplate porosity, and ultimately decrease sensory nerve innervation of porous endplates while alleviating spinal pain in mice.^24^

In clinical practice, NSAIDs are divided into two categories based on their target selectivity. Non-selective NSAIDs suppress the activity of both COX-1 and COX-2, with typical agents encompassing diclofenac, ketorolac and flurbiprofen. Selective NSAIDs specifically target COX-2 for inhibition, with representative drugs including celecoxib and etoricoxib.^72^ Multiple studies have confirmed that both non-selective and selective NSAIDs have definite efficacy in the treatment of ankle pain. For example, an open-label, multicenter randomized controlled trial (RCT) involving 278 patients with acute ankle sprains and moderate-to-severe pain showed that patients randomly assigned to take celecoxib or non-selective NSAIDs experienced relief from acute pain (assessed by Visual Analog Scale, VAS), and the efficacy of celecoxib was at least comparable to that of non-selective NSAIDs.^73^ Additionally, a prospective double-blind study (60 subjects with ankle fractures, 49 of whom completed the study) revealed that when subjects were randomly assigned to take ketorolac, diclofenac, or etoricoxib (each administered once every 12 h), ankle pain in all three groups was significantly reduced compared to baseline within 24 h (evaluated by VAS and Likert Scale), and the analgesic effects were similar across groups.^74^ However, oral NSAIDs are correlated with diverse adverse reactions affecting the cardiovascular, cerebrovascular, gastrointestinal, and renal systems. These adverse reactions also stem from COX inhibition. Among them, selective NSAIDs, by specifically targeting and inhibiting COX-2, can preserve the gastric cytoprotective effect of COX-1, thereby reducing gastrointestinal adverse reactions. Clinicians must fully consider the patient’s coexisting comorbidities when prescribing NSAIDs.^75^

Topical NSAIDs demonstrate notable efficacy in alleviating ankle pain and, in comparison with oral formulations, can more effectively avoid the common adverse events associated with oral medications.^76^ A multicenter, randomized, placebo-controlled clinical trial recruited 134 patients diagnosed with acute mild ankle sprains. Patients applied either a topical diclofenac epolamine transdermal patch (DETP) or a placebo patch daily for 7 days. The results showed that, as assessed by VAS, ankle pain was considerably reduced in the DETP group. The analgesic effect on minor sports injuries was superior to that of the placebo 4 h after drug administration, and this excellent pain relief was maintained throughout the treatment period. Furthermore, DETP can replace oral NSAIDs for the treatment of such injuries and does not exhibit the adverse event spectrum associated with oral medications.^77^ Beyond oral administration and topical application, recent studies have highlighted the application of local flurbiprofen iontophoresis to improve its local bioavailability, thus providing optimized and site-specific treatment for plantar heel pain. Research has shown that compared with oral administration and passive drug delivery, this therapy achieves higher drug levels in the plantar skin and muscles of rats and exhibits a better pharmacodynamic response to local pain.^78^

Based on the mechanism of inhibiting the COX-prostaglandin pathway, NSAIDs exert definite efficacy in the treatment of ankle pain. Different administration routes (oral administration and topical application) have their respective characteristics. Moreover, selective NSAIDs and topical application can significantly reduce systemic adverse reactions. Meanwhile, the association between NSAIDs and skeletal interoception also merits attention.

### Acupuncture for ankle pain

By stimulating specific meridians and acupoints, acupuncture can relieve pain and improve local blood circulation to facilitate functional recovery after ankle injury.^79^ Meanwhile, it can stimulate the secretion of lymphocyte inflammatory factors, reduce the accumulation of inflammatory factors caused by stress injury, improve local microcirculation, and promote the absorption of inflammatory factors as well as tissue repair and healing.^80^ A growing body of research has demonstrated that acupuncture is capable of downregulating PGE2 levels and the expression of various inflammatory factors, thus exerting pronounced anti-inflammatory and analgesic actions.^81–84^ In addition, acupuncture can exerts its therapeutic efficacy by acting on the hypothalamic-pituitary-adrenal axis and decreasing the levels of peripheral COX-2 and PGE2.^85^

Numerous studies have demonstrated that acupuncture exerts significant therapeutic effects on diseases characterized primarily by ankle pain, such as CAI and traumatic ankle osteoarthritis. For example, for ankle sprains, the effect of acupuncture at “Tongqi Ashi Acupoints” is significantly better than the external application of diclofenac diethylamine emulsion. In a RCT involving 60 patients with acute ankle sprains, both groups received basic treatment based on the modified RICE principle. After 2 weeks of treatment, the treatment group (30 cases, treated with acupuncture at “Tongqi Ashi Acupoints”) had a higher total clinical effective rate (96.67%) than the control group (30 cases, treated with external application of diclofenac diethylamine emulsion, 79.31%). Moreover, the treatment group showed better improvements in VAS scores, ankle Kofoed scores, and TCM symptom scale scores (*P* < 0.05).^86^ For CAI, the efficacy of acupuncture together with TCM external application is significantly superior to that of TCM external application alone. In a study involving 100 CAI patients, after treatment, both the control group (50 cases, treated with external application of Shenxiao Tongluo Powder) and the treatment group (50 cases, treated with external application combined with acupuncture) showed increases in AOFAS scores, average walking pressure, and gait line/footprint parameters, with the treatment group having higher values (*P* < 0.05). This confirms that acupuncture combined with external application can significantly improve ankle-foot function and plantar pressure.^87^ For traumatic ankle arthritis, the efficacy of PRP injection into the ankle joint cavity combined with warm acupuncture is significantly better than PRP injection into the ankle joint cavity alone. In a study involving 78 patients with traumatic ankle arthritis, after treatment, the treatment group (39 cases, treated with PRP injection combined with warm acupuncture) exhibited a higher overall effective rate (94.9%) than the control group (39 cases, treated with PRP injection alone, 76.9%). Additionally, the treatment group showed better VAS scores, AOFAS scores, and TCM syndrome scores (*P* < 0.05).^88^ In summary, acupuncture exerts a definite therapeutic effect in the intervention of ankle pain-related disorders, and its efficacy can be further enhanced when combined with other therapies.

### Electroacupuncture for ankle pain

Electroacupuncture is a specialized acupuncture technique that achieves preventive and therapeutic effects through minimal, low-frequency, quantifiable, and continuous electrical stimulation applied after “Deqi” (the arrival of qi, a key indicator of effective acupoint stimulation in TCM). It boasts advantages such as simple operation and long-lasting effects; besides its anti-inflammatory action, its analgesic effect is superior to that of conventional acupuncture.^89^ Consistent with acupuncture, electroacupuncture is also capable of exerting anti-inflammatory and analgesic effects by lowering the concentrations of PGE2 and multiple pain and inflammatory factors.^90–92^

In clinical practice, electroacupuncture has also demonstrated favorable efficacy in treating conditions like ankle sprains. For instance, it is more effective than ultrasound therapy in treating ankle sprains and CAI. A RCT involving 60 patients with ankle sprains included two groups, both receiving basic treatment of splint fixation and functional exercise. The treatment group (30 cases) underwent electroacupuncture, while the control group (30 cases) received ultrasound therapy, with 3 treatment sessions per week for 2 weeks. Following treatment, the overall effective rate of the treatment group (92.85%) was markedly higher than that of the control group (75%). Moreover, the treatment group showed better results in terms of the Young’s modulus of the anterior talofibular ligament, scores on the AOFAS scale, and VAS scores (*P* < 0.05).^93^ These findings indicate that electroacupuncture can effectively relieve pain from ankle sprains, improve joint function, and is more effective than ultrasound therapy. Furthermore, electroacupuncture can effectively improve static balance ability in patients with CAI classified by different Hiller types. In a RCT, researchers enrolled 140 CAI patients (with 26 cases lost to follow-up eventually). All patients were grouped according to the Hiller classification criteria and then treated with electroacupuncture, with the therapeutic regimen set as twice a week for 4 consecutive weeks. Static balance stability was assessed using the 30-second single-leg standing test with eyes closed, star excursion balance test, and single-leg side alternate jump test before and after treatment. The results demonstrated that all balance indicators of patients in each group were significantly improved after treatment compared with baseline (*P* < 0.001).^94^ Therefore, with its definite therapeutic effects, electroacupuncture can serve as one of the effective treatment options for ankle pain-related disorders.

### Other traditional chinese medicine (TCM) therapies for ankle pain

In TCM theory, acute soft tissue injury is categorized under “shangjin”, “jinshang”, and “bizheng”. Its core pathogenesis lies in collateral damage caused by qi stagnation and blood stasis, as well as disharmony of collaterals.^95^ TCM therapies such as oral TCM, TCM fumigation and washing, TCM external application are commonly used for treating ankle pain and other related conditions. Recent studies have confirmed that these internal and external therapies, with external therapies in particular, exert therapeutic effects by reducing PGE2 levels. For example, heat-clearing and blood-stasis-resolving Chinese herbs and herbal ointments, such as Babu Ointment, can effectively lower PGE2 levels in rabbits with acute soft tissue injury models and promote the resolution of swelling and ecchymoses.^95,96^ When Huayu Qushi Formula is administered for knee osteoarthritis of phlegm-dampness and blood-stasis syndrome, or when electroacupuncture or extracorporeal shock wave combined with TCM fumigation and washing is applied for lumbar-related diseases, PGE2 levels can be downregulated. In some cases, this approach also reduces levels of other indicators such as MMP-13 and IL-6, thereby alleviating pain and improving joint function.^97–99^ Notably, TCM internal and external therapies, especially external ones, exhibit significant efficacy in treating acute soft tissue injury. The medicines act directly on the lesion site, reducing the metabolic burden on the liver and kidneys, and greatly lowering toxicity to the gastrointestinal tract, kidneys, and heart.^95^

### Surgical interventions for ankle pain

For AOA, conservative treatment can alleviate pain and potentially maintain joint function in the early stages.^100^ However, in the advanced stages, AOA triggers severe ankle pain and functional impairment, which drastically compromise patients’ quality of life. At present, ankle arthrodesis and total ankle arthroplasty are the most commonly adopted surgical interventions for this condition.^101^

Ankle arthrodesis has long been recognized as a classic surgical approach for the treatment of diverse ankle disorders. It is indicated for patients diagnosed with RA, post-traumatic arthritis, infectious diseases, neuromuscular diseases, and those with failed total ankle arthroplasty. This procedure remains the first choice especially for patients who require long-term participation in high-intensity activities.^102^ The surgery involves complete resection of articular cartilage, proper compression and stable fixation of the fusion site, and relies on standardized postoperative rehabilitation programs and satisfactory patient compliance to achieve successful joint fusion.^103^ Nonetheless, this surgical method carries certain disadvantages, including nonunion of the fused joint, long-term immobilization after surgery, and a high risk of long-term degenerative arthritis in adjacent joints.^104^ One study confirmed that ankle arthrodesis with anterior or lateral locking plate internal fixation achieves favorable clinical efficacy in the treatment of post-traumatic AOA, promotes the recovery of ankle function, and further reveals that PGE2 levels are reduced one week after surgery.^105^

Total ankle arthroplasty is also an effective alternative for the management of end-stage AOA.^104^ This procedure achieves the core therapeutic goal of pain relief while preserving the mobility of the ankle joint.^106^ Nevertheless, total ankle arthroplasty is technically demanding with a steep learning curve, and the long-term survival rate of the prosthesis has not been fully clarified.^104^ Comprehensive preoperative evaluation is essential, covering patients’ ankle range of motion, severity of coronal plane deformity, and baseline activity level, to guarantee satisfactory postoperative outcomes.^107^ A systematic review and meta-analysis has demonstrated that total ankle arthroplasty exerts positive impacts on the quality of life, daily activities and motor function recovery of patients with orthopedic ankle injuries.^108^ In addition, total ankle arthroplasty can effectively reduce the intra-articular level of PGE2 in the short term by resecting local inflammatory lesions such as synovial hyperplasia and degenerative cartilage, thereby directly eliminating the synthetic source of this inflammatory mediator.^109^

Notably, compared with ankle arthrodesis, total ankle arthroplasty significantly lowers the risks of postoperative infection, amputation and joint fusion failure, and better preserves the overall mobility of the ankle joint in patients.^110^ However, the revision rate of total ankle arthroplasty is notably higher than that of ankle arthrodesis. Therefore, careful patient selection is critical to ensure successful treatment of end-stage AOA. Ankle arthrodesis and total ankle arthroplasty have their own strengths and weaknesses, and the surgical plan should be determined based on patients’ treatment expectations and individual characteristics, including bone and soft tissue quality, age, diabetes, valgus tilt and medial laxity.^100^

## Conclusions

In this review, we highlight the concept of skeletal interoception, which specifically refers to the perception and processing of skeletal signals. This process is crucial for maintaining skeletal homeostasis and effectively responding to mechanical loading. While existing studies have delineated the research advances in skeletal pathology and pain, the present review synthesizes these findings to specifically highlight the functional interplay between PGE2 and skeletal interoception amid pain and inflammatory contexts. Additionally, within the framework of the skeletal interoception/PGE2 pathway, we elucidate the mechanistic links between the pathogenesis of ankle pain and diverse therapeutic strategies, including NSAIDs. However, a notable limitation of current research lies in the relative paucity of studies investigating the correlation between skeletal interoception and the pathogenic mechanisms of ankle pain. Looking ahead, building on recent advancements in skeletal interoception research, we can further investigate the pathogenesis and therapeutic mechanisms underlying ankle pain. Such endeavors may yield novel insights into the clinical management of ankle pain, ultimately improving treatment outcomes for affected patients.
